# Gold Standard Evaluation of an Automatic HAIs Surveillance System

**DOI:** 10.1155/2019/1049575

**Published:** 2019-09-23

**Authors:** Beatriz Villamarín-Bello, Berta Uriel-Latorre, Florentino Fdez-Riverola, María Sande-Meijide, Daniel Glez-Peña

**Affiliations:** ^1^Preventive Medice Service, Complexo Hospitalario Universitario de Ourense, Rúa Ramón Puga 52-56, 32004 Ourense, Spain; ^2^Department of Computer Science, University of Vigo, ESEI—Escuela Superior de Ingeniería Informática, Edificio Politécnico, Campus Universitario As Lagoas s/n, 32004 Ourense, Spain; ^3^CINBIO—Biomedical Research Centre, University of Vigo, Campus Universitario Lagoas-Marcosende, 36310 Vigo, Spain; ^4^SING Research Group, Galicia Sur Health Research Institute (IIS Galicia Sur), SERGAS-UVIGO, Vigo, Spain

## Abstract

Hospital-acquired Infections (HAIs) surveillance, defined as the systematic collection of data related to a certain health event, is considered an essential dimension for a prevention HAI program to be effective. In recent years, new automated HAI surveillance methods have emerged with the wide adoption of electronic health records (EHR). Here we present the validation results against the gold standard of HAIs diagnosis of the InNoCBR system deployed in the Ourense University Hospital Complex (Spain). Acting as a totally autonomous system, InNoCBR achieves a HAI sensitivity of 70.83% and a specificity of 97.76%, with a positive predictive value of 77.24%. The kappa index for infection type classification is 0.67. Sensitivity varies depending on infection type, where bloodstream infection attains the best value (93.33%), whereas the respiratory infection could be improved the most (53.33%). Working as a semi-automatic system, InNoCBR reaches a high level of sensitivity (81.73%), specificity (99.47%), and a meritorious positive predictive value (94.33%).

## 1. Introduction

Hospital-acquired (nosocomial) infections are defined as infections contracted in a hospital environment being not present, nor in the incubation period, at the inpatient admission date [1]. Following this definition, it is commonly accepted that those infections occurring after the first 48 h from the hospital admission date are considered as hospital-acquired infections (HAIs). In Europe, it is estimated that 4,100,000 patients suffer from any type of nosocomial infection every year. In this context, the EPPS (European Point Prevalence Survey) showed that, in the 2011-2012 period, the prevalence of patients with at least one HAI in acute care hospitals was 6.0%, which means that 1 from 18 admitted patients suffer one HAI every day. This prevalence increases to 19.5% in intensive care patients. According to WHO (World Health Organization) data, 37,000 deaths are directly related to HAIs and up to 16 million of avoidable hospital admissions take place in Europe every year.

The surveillance, defined as the systematic collection of data related to a certain health event, is considered an essential dimension for a prevention HAI program to be effective [2, 3]. In such a situation, surveillance activities are a first step towards HAI prevention, showing that through the implementation of appropriate surveillance and control programs, a reduction of up to 20%–30% in the occurrence of HAIs can be achieved [4–6]. As part of this approach, traditional surveillance is based on time-consuming manual inspections, which require (i) daily revision of lengthy lists containing micro-organisms found in positive cultures from the microbiology service and drug prescriptions from the pharmacy service, (ii) regular visits to the medical inpatient units, (iii) revision of the clinical histories (e.g., evolution records, annotations from nursing staff, analytical data, etc.,), and (iv) compute the necessary calculations for estimating the infection rate. All these activities should be done in advance, within a reasonable short time, in order to establish appropriate corrective actions where necessary in the most quick and efficient manner. While the benefits of this traditional real-time surveillance are undeniable, this mode of operation is expensive and difficult to assume for the vast majority of the preventive medicine services, which often see other relevant activities seriously undermined.

However, new automated HAI surveillance methods have emerged with the wide adoption of electronic health records (EHR). These systems facilitate the daily work of surveillance while, at the same time, improve the effectiveness and efficiency of the whole process, allowing the monitoring of large hospital areas with an optimum use of available resources. For example, Du et al. [7] developed and validated RT-NISS, a real-time automatic hospital-wide HAIs surveillance system in China. The validation over 974 (85 HAI and 889 nonHAI cases) manually checked inpatients gave excellent rates of sensitivity (98.8%) and specificity (93.0%), and a more modest positive predictive value (PPV) (57.53%). They also report time savings against manual review of 200 times. Tvardik et al. [8] studied the feasibility of using Natural Language Processing techniques to automatically detect HAIs in clinical documents. They tested the system over 113 cases (56 HAI and 57 nonHAI cases) and obtained a sensitivity of 83.9% and a specificity of 84.2%. The PPV was not reported, but in a real setting, where the prevalence of HAIs (or proportion of positive cases) is relatively low (about 6%), the reported sensitivity and specificity would lead to a high false discovery rate, or low PPV. A review of automated surveillance of HAIs can be found in [9].

The InNoCBR system is an automatic HAI detection and classification software developed between 2010 and 2013, and is routinely used at the Preventive Medicine Service of CHUO (Ourense University Hospital Complex, Spain), a public hospital belonging to the Spanish National Health System. During all that time, the system was systematically applied to monitor, diagnose, and control HAIs under the supervision of infection control specialists following the well-known surveillance definitions and criteria adopted by the ECDC (European Centre for Disease Prevention and Control). InNoCBR is able to detect and classify HAIs of multiple types including urinary, respiratory, bloodstream, surgical site, cutaneous, enteric, and other type. The system was described and partially validated in [10], but only with those cases that were automatically gathered with an acquisition process inside InNoCBR. In this sense, validation in [10] focused in the ability of InNoCBR to correctly learn from the user (expert) behavior when classifying the correct type of HAI of a suspicious case (by means of Machine Learning techniques). In this sense, in [10] we could not, for example, assess false negatives i.e.,: HAI cases that were not acquired. Moreover, the “gold standard” used in [10] was the InNoCBR user classifications by seeing the patient information gathered by InNoCBR.

Here we present the validation results of the whole InNoCBR system against the gold standard of HAIs diagnosis, i.e., the manual review of every possible case carried out by independent experts.

## 2. Materials and Methods

### 2.1. InNoCBR

**Figure 1:**
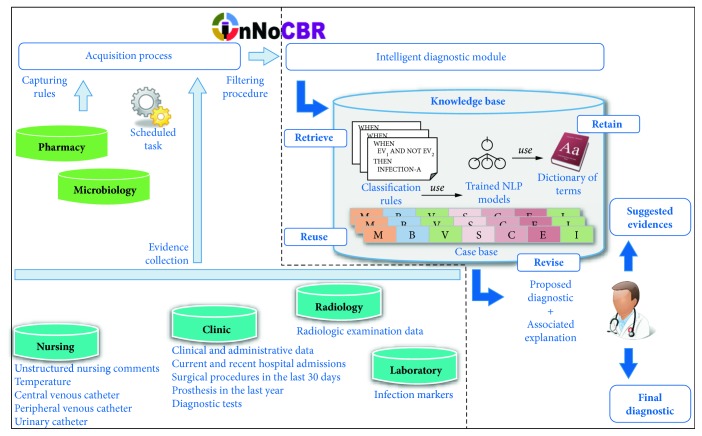
InNoCBR system overview: application architecture and operational process.

Taking into consideration that the appropriate identification of HAIs involves the selection of an initial manageable (but highly sensitive) subset of potentially positive cases from the entire patient database, InNoCBR was divided into two well-differentiated operational modules executed by a scheduled task on a daily basis: (i) gathering of potentially positive HAI cases (namely, the acquisition process), and (ii) the intelligent diagnostic module itself.  Figure 1 summarizes the InNoCBR architecture and the underlying operational process. The main objective of the first (left side) module is the identification of possible HAI cases irrespective of their location while optimizing sensitivity (i.e., preventing the existence of false negative errors). For its part, the second (right side) module is in charge of executing the intelligent diagnostic process, in which those previous collected cases are classified by infection type taking into consideration evidence found in the hospital information systems.

On the one hand, the acquisition process (left side module in Figure 1) is carried out using two different but complementary sources of information (i.e., databases of microbiology and pharmacy), from which several sets of capturing rules and filters are applied with the goal of discarding those less promising cases. In practice, cases from the microbiology database are selected when positive samples of a certain micro-organism are found in cultures from admitted patients, or patients coming from the emergency department or external consultations. In a complementary action, the pharmacy database is used to find antimicrobial prescriptions with a duration longer than 5 days, selecting those patients as potentially positive HAI cases. Additionally, each single period of hospitalisation may result in several potentially positive HAI cases, as in the case of patient prescriptions being interrupted over one or more days, which are considered as a separate HAI case.

During the application of the set of capturing rules previously commented, different kinds of complementary information (labelled as evidence collection in Figure 1) are also stored for the subsequent phase of filtering and the later execution of the intelligent diagnostic module. This additional knowledge comprises (i) administrative patient data, (ii) surgery done in the prior month analysed, (iii) prosthesis placed in the last year, (iv) hospital admissions in a time window around the analysed period, (v) departments and hospital beds for which a patient goes through including the nursing unit in case of positive micro-organism cultures, (vi) the existence or not of radiological reports, (vii) unstructured nursing comments, (viii) general observations from the microbiology report, (ix) additional annotations about fever, leucocyte presence in urinary samples, and (x) presence of central and peripheral catheters, and/or urinary catheters.

As previously stated, the primary goal of the acquisition process is the identification of all the possible HAI cases (using capturing rules to maximize sensitivity), which usually leads to a large number of potential candidates: many of them generated by the same infection process and the rest being false positives. In order to counteract this situation, InNoCBR applies a filtering procedure which depends on the specific database used as original source of information (i.e., microbiology or pharmacy).

The specific filtering rules used for those cases coming from the microbiology database are the following: (i) positive blood cultures of the same day for a given patient only generate a potentially positive HAI case belonging to the first positive blood culture, (ii) positive blood cultures and any other sample related with the same micro-organism in a time window of 4 days (back and forth), only generate a unique potentially positive HAI case linked to the sample which is not of blood, (iii) considering a time window of 10 days, positive cultures of the same sample and the same micro-organism (even if there are more than one micro-organism in the sample) only generate a unique potentially positive HAI case, (iv) positive samples of nasal exudate and catheter tip are not considered as potentially positive HAI cases, (v) positive samples (not related to hospitalisation) belonging to patients who did not undergo surgery in the previous month or prosthesis in the last year, are not taken into account, (vi) positive cultures within the first two days after hospitalisation are not considered unless they are exudate, in which case it will be checked whether the patient underwent surgery or prosthesis, and (vii) positive samples of bronchial suction or bronchoalveolar lavage belonging to outpatients from external consultations are not considered.

In respect of the filtering rules used for those cases coming from the pharmacy database (characterized by the prescription of an antibiotic), InNoCBR takes the following into consideration: (i) if a sample from the microbiology database exists in a time window of 4 days (back and forth) from the day of initiation of the given antibiotic treatment, this case will not be considered as a potentially positive HAI, (ii) samples from the pharmacy database whose day of initiation of the antibiotic treatment falls between the first 2 days of the clinical event will not be considered as potentially positive HAI cases, (iii) samples from the pharmacy database that do not have any radiological report nor positive leucocytes associated in a time window of 4 days (back and forth) from the starting date of the acquisition process are automatically discarded, and (iv) if the difference between the acquisition date of two samples coming from the pharmacy database is 4 days or less, the latter is excluded.

On the other hand, the intelligent diagnostic module (right side in Figure 1) automatically classifies every potentially positive HAI case (gathered in the previous acquisition phase) as one of the following categories: (i) HAI, including its location, (ii) extrahospitalary infection, or (iii) no infection. The development of the intelligent classification model was the result of a previous doctoral thesis titled “*Intelligent system for searching and classification of nosocomial infection cases*” [11] whose main contributions can be found in [10]. In summary, the intelligent diagnostic module is based on a CBR (Case-Based Reasoning) system [12], equipped with (i) a set of manual rules provided by experts focused on urinary, surgical, and bloodstream infection types and (ii) a set of automatic extracted rules (AER) that deal with samples that are not classified by the manual rules that were derived by using the PART Machine Learning algorithm [13]. Additionally, a NLP (Natural Language Processing) unit is able to handle precise electronic physician narratives and daily comments from nursing staff in order to provide additional clues about the occurrence of underlying HAIs.

### 2.2. Gold Standard

The gold standard for the comprehensive evaluation of the InNoCBR system was based on the collection and storage of data from periodic visits to patients admitted to six selected units, which included both (i) gathering individual comments about the patient follow-up from the nursing staff, and (ii) the direct observation of certain relevant symptoms. All the data gathered by means of these on-site visits were the complement to the information provided through EHR, which included data coming from microbiology results, analytical determinations, imaging studies, physician narratives, and comments from nursing staff, previous admissions and information from primary care services.

Each of the inpatient units participating in the study was analysed during a time frame of one month, although the follow-up time of each individual patient was not equal for all. In case of patients already admitted in the unit during the beginning of the study, the monitoring was carried out from the inpatient admission date to hospital discharge, patient transfer to another unit or the very last day of the study. In addition, those patients who were admitted in the unit once the study was started only were monitored until their hospital discharge, transfer to another unit or the very last day of the study.

Taking into consideration all the information collected and structured during the entire period (i.e., the gold standard), an infection control specialist made a final diagnosis for each analysed patient choosing one of three available categories: (i) HAI, (ii) extrahospitalary infection, or (iii) no infection.

### 2.3. Experimental Design

The present work is a validation study of a diagnostic test, and therefore a descriptive, comparative, and transversal study was particularly designed to adequately compare the output generated by an autonomous HAIs surveillance system against the gold standard. As in previous related works form the scientific community, standard measures were used to validate the accuracy and overall performance of our InNoCBR system including sensitivity, specificity, and both positive predictive and negative predictive values. Additionally, the prevalence of nosocomial infection was adjusted by taking into account its value from the EPINE (Study of Prevalence of Nosocomial Infection in Spain) in 2012 [14].

With regard to the population under study and the period analysed, there have been studies on all the patients admitted in the following units during the period specified: (i) traumatology unit in April 2013, (ii) internal medicine unit in August 2013, (iii) intensive care unit (ICU) including in September 2013, (iv) nephrology unit in February 2014, (v) general surgery unit from the 19th March to 20th April 2014, and (vi) reanimation unit from 19th March to 20th April 2014.

In reference to inclusion and exclusion criteria, all the patients admitted in the units mentioned above during each analysed period were included. As a result, a large number of patients were selected with the goal of representing the whole spectra of infection. Throughout the period analysed, some patients suffered a HAI while they were hospitalised while others had no symptoms of infection. In a complementary way, some patients have been excluded from the present study because they were admitted to different units or they did not fulfil the time criterion of the target units.

In relation to the population size, a lower limit was not previously established but a representative set of hospital units were initially selected in order to cover a balanced representation of the disease spectrum over a sufficient period of time. In practice, this resulted in a total of 890 patients distributed as follows: 221 patients from the traumatology unit, 169 patients from the internal medicine unit, 104 patients from the ICU, 43 patients from the nephrology unit, and 353 patients from the general surgery and reanimation units, which were studied as a whole because after a surgical intervention, the patient often remains in the reanimation unit for some time.

With respect to evaluation measures, they can be derived from confusion matrixes. For a prediction system for two conditions (for example discriminating between HAI and nonHAI cases), a 2 × 2 confusion matrix as the one shown in Table 1 is used, where there can be four types of results: true positives (TP) which are positive cases predicted as such, false positives (FP), which are negative cases predicted as positives, false negatives (FN) which are positive cases predicted as negative, and true negatives (TN) which are negative cases predicted as such.

From this matrix, the sensitivity (Se), as the proportion of positive cases correctly identified, specificity (Sp), as the proportion of negative cases correctly identified, positive predictive value (PPV), as the of positive predictions that are correct and negative predictive value (NPV), as the proportion of negative predictions that are correct, can be calculated with the following formulas:

(1) $$\begin{array}{c} \text{Se}=\frac{\mathrm{TP}}{\text{TP}+\text{FN}},\text{Sp}=\frac{\mathrm{TN}}{\text{TN}+\text{FP}},\text{PPV}=\frac{\mathrm{TP}}{\text{TP}+\text{FP}},\text{NPV}=\frac{\text{TN}}{\text{FN}+\text{TN}}. \end{array}$$

For a prediction system that classifies cases between *C* different conditions (for example classify the correct type of HAI), a *C *× *C* confusion matrix as the one shown in Table 2 can be calculated. Each *i*, *j* cell contains the count of cases presenting condition *j* predicted as having condition *i*.

The Cohen's kappa index is calculated given a *C* × *C* confusion matrix by the following formula:

(2) $$\begin{array}{c} K=\frac{\sum Xii-\sum XiXi}{1-\sum XiXi}, \end{array}$$

where *i* takes values from 1 to *C*.

For cases where the true conditions in the obtained gold standard have a different distribution, that is, the real population prevalence differs from the sample observed one, some measures need to be adjusted for real prevalence. Prevalence-adjusted PPV and NPV values are calculated with the following formulas (prev = population prevalence):

(3) $$\begin{array}{c} \text{PPV}=\frac{\text{Se}*\text{prev}}{\text{Se}*\text{prev}+\left(1-\text{prev}\right)*\left(1-\text{Sp}\right)},\text{NPV}=\frac{\text{Sp}*\left(1-\text{prev}\right)}{\text{prev}*\left(1-\text{Se}\right)+\text{Sp}*\left(1-\text{prev}\right)}. \end{array}$$

In the multiple-condition case, the adjustment is done by redefining the C × C confusion matrix values as:

(4) $$\begin{array}{c} \text{adjusted}\left(X_{\mathrm{ij}}\right)=\frac{X_{\mathrm{ij}}}{\sum_{i=1}^{C}X_{ij}}\cdot {\text{prev}}_{j}, \end{array}$$

where prev_*j*_ is the true prevalence for condition *j*.

## 3. Results and Discussion

Four different sides of InNoCBR were evaluated, including (i) the InNoCBR acquisition process, as the capability of detecting HAIs regardless from its concrete type of infection, measuring sensitivity, specificity, PPV, and NPV, (ii) the InNoCBR intelligent diagnostic process, as the capability to correctly classify those suspicious HAI cases coming from the acquisition module, measuring sensitivity, specificity, PPV, NPV, as well as, Cohen's kappa index for the concrete type of infection agreement, (iii) the global combination of the previous two modules, evaluating the whole automatic HAI detection and classification system as a whole, measuring sensitivity, specificity, PPV, NPV, as well as, Cohen's kappa index for the concrete type of infection agreement, and (iv) the performance as a semi-automatic system, that is, the comparison of the final decision of the InNoCBR user against the gold standard, instead of the InNoCBR's diagnostic proposal.

### 3.1. Gold Standard

Gold standard fieldwork resulted in a valuable final raw data set comprising 938 possible HAI cases belonging to the 890 patients that conform the population under study in the analysed period. Each patient was identified by its own medical record number, which was subsequently used to review all the information derived from the inpatient hospitalisation with the goal of reaching a consensual diagnosis (i.e., HAI and particular type of infection, extrahospitalary infection or no infection).  Table 3 shows the absolute number of cases comprising the gold standard by each analysed unit and type of infection.

From Table 1, as would be expected, it may be observed that the greatest number of surgical site infections (S) occur in the general surgery and reanimation units. Even though, it is also notable the number of urinary infections in these same units, that might be related to the vesical catheterization used by patients in the first hours our days postoperatively.

Additionally, it is also noted that urinary infections are the most common in all the hospital units analysed, which is in line with the study of prevalence of nosocomial infection in Spain (EPINE), being justified by the frequent use of vesical catheters in admitted patients. In respect of respiratory infections, a large number of occurrences is observed in the ICU, which is very probably linked to the inpatient population admitted in this unit, almost all of them requiring mechanical ventilation or being in close contact witch patients with respiratory infections.

The validation of the InNoCBR system compared with the gold standard is addressed as a validation study of a diagnostic test and, therefore, it is of special interest to obtain accuracy and overall performance results in terms of validity and security. In this line, standard measures were used to validate the system including sensitivity, specificity, positive predictive value, negative predictive value, and kappa for its two operational modules (i.e., acquisition process and intelligent diagnostic) working separately and together. In this regard, Table 4 presents the global confusion matrix summarizing the classification results obtained following the experimental design protocol previously commented.

Interestingly, the global confusion matrix presented in Table 4 contains the number of those cases not acquired by the InNoCBR acquisition module (indicated in parentheses). As an example, it may be observed that there are a total of 78 urinary infections, from which 10 are not acquired by the InNoCBR acquisition module, while the remaining are correctly classified as urinary (60) or incorrectly classified as negative by the intelligent diagnostic module. Moreover, it may also be observed that numerous discrepancies take place when a specific type of infection is classified as a negative case, except for two infections of type “O” that were classified as surgical infections by InNoCBR.

### 3.2. InNoCBR Acquisition Module

As previously commented, the acquisition process of InNoCBR is carried out using different information coming from the microbiology and pharmacy databases, with the goal of gathering potentially positive HAI cases. This section presents the results obtained by the system with regard to its ability to detect actual HAI cases, irrespective of their location. In this line, Table 5 summarizes the results obtained by the InNoCBR acquisition module when compared with the gold standard.

The results obtained show an acceptable level of sensitivity (88.76%), which is the main objective of the acquisition module (i.e., preventing the existence of false negative errors). This value, together with the achieved specificity level and also considering a prevalence value of HAI equals to 9.68%, produces a modest result for PPV (46.26%). This fact highlights the need for the subsequent intelligent diagnostic module, which is in charge of further analysing those previous collected cases to classify true positives by infection type and discard all the false negatives previously collected.

In reference to the acquisition process, in particular when considering the infection type, Table 6 summarizes the results obtained by the acquisition module of InNoCBR.

It is important to note that the number of cases with cutaneous infection, enteric, and other locations is lower than 10, therefore, data are not conclusive in this respect. As for the remainder of the locations in descending order of achieved sensitivity, the following values were obtained: surgical site = 97.78%, bloodstream = 93.33%, urinary = 87.18% and respiratory = 80%. Since the respiratory infection is significantly lower compared to the rest of HAIs, it should be considered as an improvement point of the InNoCBR acquisition process.

### 3.3. InNoCBR Intelligent Diagnostic Module

Once the acquisition module selects all the potentially positive HAI cases, InNoCBR automatically executes the intelligent diagnostic module. This section presents the results obtained by this module taking only into account those previously acquired cases.  Table 7 summarizes the results obtained by the InNoCBR intelligent diagnostic module when compared with the gold standard.

As shown in Table 7, both sensitivity and specificity values are around 80%. PPV is much higher than in the acquisition module, reaching a percentage of 77.24%. Additionally, a moderate kappa value of 0.62 is obtained by the intelligent diagnostic module.

### 3.4. Global Performance of the InNoCBR System

In order to obtain a comprehensive and aggregate view of the InNoCBR system (i.e., acquisition + intelligent diagnostic modules working as a whole), it can be evaluated following a *black box* approach, without examining each part separately. In this connection, Table 8 shows the same performance measures as before, but considering the system as a whole.

As shown in Table 8, the global sensitivity value of InNoCBR is 70.83%, which is less than the value obtained for the acquisition or intelligent diagnostic modules analysed separately. This can be explained by the fact that some correctly acquired HAI cases, are latter discarded by the intelligent diagnostic module. Moreover, in no case the intelligent diagnostic module can improve the acquisition module, because it is impossible to re-acquire any additional HAI case. For its part, the specificity level achieves a 97.76%, higher than the value obtained by the separate modules. The PPV value (77.24%), as would be expected, is the same as in the intelligent diagnostic module and, therefore, significantly higher than the value obtained by the acquisition module. Finally, the Kappa index achieves a moderate value of 0.67.

**Figure 2:**
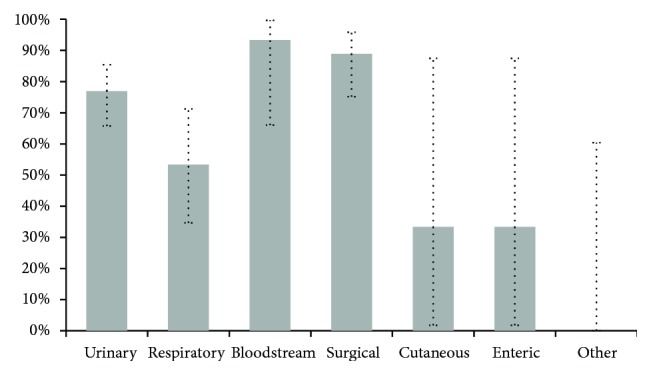
Global sensitivity of InNoCBR when detecting possible HAI cases from different locations. Vertical error bars indicate potential variations with 95% confidence intervals.

To complement the global performance study of the InNoCBR system, Figure 2 shows a sensitivity analysis by infection type.

As shown in Figure 2, and omitting those cases with cutaneous infection, enteric, and other locations because of the low number of instances, obtained sensitivities in descending order were bloodstream infection = 93.33, surgical site = 88.89%, urinary = 76.92%, and respiratory = 53.33%. Once again, the respiratory infection should be considered as an improvement point.

**Figure 3:**
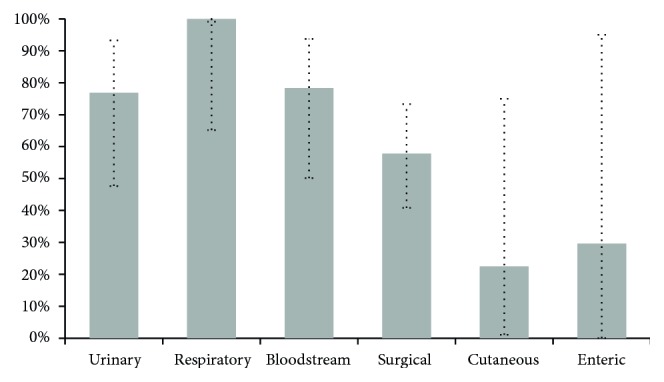
Global PPV of InNoCBR when detecting possible HAI cases from different locations. Vertical error bars indicate potential variations with 95% confidence intervals.

With reference to PPV taking into consideration different locations (i.e., reliability of a positive case), Figure 3 shows the results obtained by the InNoCBR system. In this case, the infection labelled as “Other” was not included because there was no such output from the InNoCBR system. As in previous analyses, the low number of cases for cutaneous and enteric locations led to inconclusive results. As for the remainder of the locations in descending order of achieved VPP, the following values were obtained: respiratory = 100.00%, bloodstream = 78.32%, and urinary = 76.87%. Specifically, a high reliability was obtained for respiratory HAIs, because InNoCBR correctly diagnosed all the available cases. However, the number of positive predictions still offers only a limited scope to draw definite.

**Figure 4:**
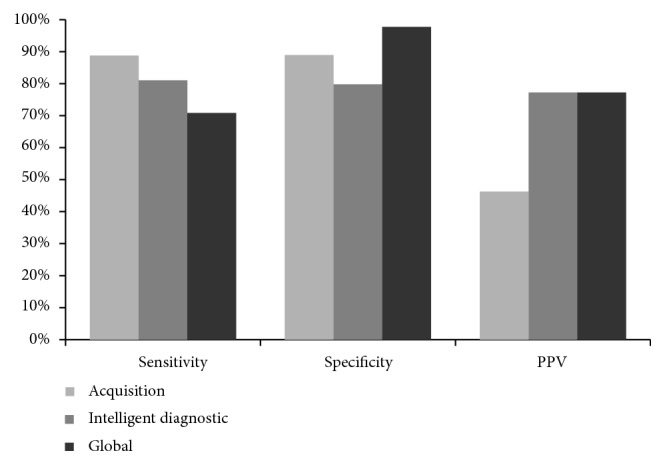
Global sensitivity, specificity and PPV values obtained by the InNoCBR system, disaggregated by the acquisition and intelligent diagnostic modules. Note: both VPP of the intelligent diagnostic module and VPP of the InNoCBR system have the same value by definition.

By way of summary, Figure 4 presents the collaborative work between the two InNoCBR modules, working in a co-ordinated manner to obtain results in two key aspects: sensitivity and PPV. In this way, the InNoCBR acquisition module exhibits a high sensitivity percentage at the expense of a high false positive rate (resulting in a low PPV), but the intelligent diagnostic module obtains a much lower false positive rate while keeping an acceptable sensitivity. In this context, it is important to note that the loss in sensitivity shown by the intelligent diagnostic module has a smaller impact than if it would have taken place in the acquisition module, as all the acquired cases are also analysed by the expert, whereas if they are not acquired, they are definitively lost (not been counted).

### 3.5. InNoCBR Performance Working as a Semi-Automatic Diagnostic System

To complement the validation study, this section analyses the performance of InNoCBR working as a recommendation system for the purpose of assisting the expert with the final diagnosis. In this particular case, the comparison is made between the final diagnostic of the expert (with the assistance of InNoCBR) vs. the gold standard, which can be seen as a global assessment of the Preventive Medicine Service equipped with an automatic tool for the purpose of detecting and classifying HAIs. In this scenario, it must be pointed out that the expert only evaluates those cases previously acquired by InNoCBR.  Table 9 presents the new confusion matrix corresponding to InNoCBR working as a semi-automatic diagnostic system.

On the basis of the confusion matrix introduced in Table 9, Table 10 summarizes the results obtained by InNoCBR working as a semi-automatic diagnostic system when compared with the gold standard.

As shown in Table 10, the global sensitivity value of InNoCBR working as a semi-automatic diagnostic system reaches an 81.73%. This value has a theoretical ceiling of 88.76% derived from the acquisition module of InNoCBR, since the expert does not evaluate any cases that were not previously acquired. In this respect, and as previously mentioned, sensitivity is the measure that should be improved, mainly in the acquisition module. For its part, the specificity level achieves a 99.47%, motivated by the fact that the expert has rectified 80 samples out of 84 negative cases incorrectly acquired. The PPV value attains a 94.33% and the NPV value remains in a 76.00%. Table 8 also includes the Kapa index for those acquired cases that are evaluated by the expert, obtaining an optimum value of 0.91.

**Figure 5:**
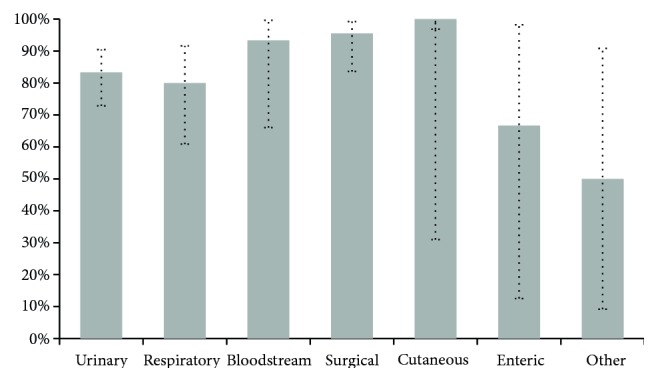
Sensitivity of InNoCBR working as a semi-automatic diagnostic system when detecting possible HAI cases from different locations. Vertical error bars indicate potential variations with 95% confidence intervals.

To complement this section, Figure 5 shows the sensitivity analysis of InNoCBR working as a semi-automatic diagnostic system by infection type. From Figure 5, and omitting those cases with cutaneous infection, enteric, and other locations because of the low number of instances, it may be observed that obtained sensitivities in descending order were surgical site = 95.56%, Bloodstream = 93.33, urinary = 83.33%, and respiratory = 80.00%. Based on this information, the semi-automatic diagnostic system clearly improves sensitivity values in all cases, and in particular in the respiratory HAI.

**Figure 6:**
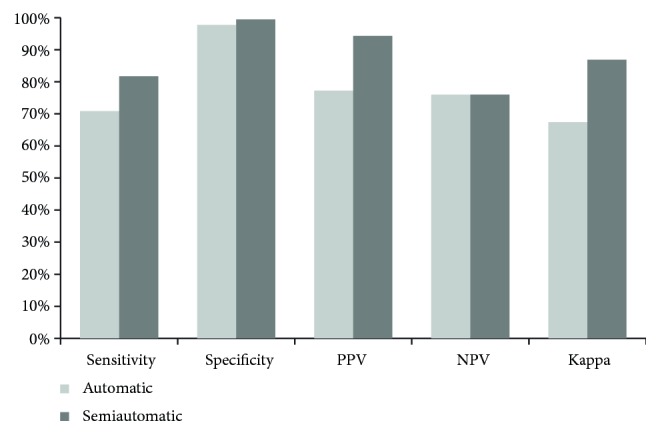
Sensitivity, specificity, PPV, NPV and kappa obtained by InNoCBR (working as an automatic and semi-automatic system) VS gold standard.

Finally, Figure 6 summarizes different performance measures that evidence the improvement achieved by InNoCBR working as a semi-automatic diagnostic system when compared with its intelligent diagnostic module working individually.

### 3.6. Errors Analysis

Among the 63 errors committed by InNoCBR, there are seven types of discrepancies. In the acquisition process, whose objective is to be highly sensitive, only false negatives can be considered as errors, because a false positive can be corrected by the intelligent diagnostic process that run afterwards. Concretely, a false negative in the acquisition module can be due to (i) lack of acquisition from microbiology or pharmacy databases, (ii) erroneous filtering of a sample acquired from the pharmacy database, or (iii) erroneous filtering of a sample acquired from the microbiology database. With respect to the intelligent diagnostic process, the type of errors include (i) samples acquired from pharmacy are not automatically classified since this feature is not implemented in InNoCBR, (ii) incorrect type of HAI, (iii) false positive, which is a nonHAI that was erroneously predicted as HAI, and (iv) false negative, which is a HAI that was erroneously identified as nonHAI.  Table 11 Summarizes these different types of discrepancies found during validation of InNoCBR.

As can be observed in Table 11, an important number of errors are produced by erroneous filters (18). We observed that all erroneously filtered microbiology samples were filtered with the following filter (see materials and methods): *positive cultures within the first two days after hospitalisation are not considered unless they are exudate, in which case it will be checked whether the patient underwent surgery or prosthesis*. In those cases a previous surgery or a previous hospitalisation was present, but the sample was not exudate. It could be interesting to consider more different types of samples in this filter rule. We also observed that some erroneously filtered pharmacy samples were due to the following filter: *samples from the pharmacy database whose day of initiation of the antibiotic treatment falls between the first 2 days of the clinical event will not be considered as potentially positive HAI cases*. In some cases, those patients had undergo a recent surgical procedure or admission, so an exception in this sense for this filter could be considered. Another important source of errors is the fact that InNoCBR does not process samples acquired from pharmacy (15 errors), being an evident source of false negatives. Finally, false positives and negatives (17 and 9 errors, respectively) in diagnostic module where produced in samples processed with manually expert-provided rules (15/128 cases) or in samples processed with the automatically extracted rules (13/63). Expert-provided rules have more accuracy than automatically extracted ones, as it could be expected. In this sense, it could be interesting to incorporate manual rules focused on respiratory infections, which showed the lowest sensitivity. However, experts indicated that it would be necessary to access medical comments in RX reports, which was not possible due to technical restrictions related with hospital information systems access.

## 4. Conclusions

Here we have presented the evaluation of an automatic HAI detection and classification system, InNoCBR, which was routinely used at the Preventive Medicine Service of the Ourense University Hospital Complex from 2013. The validation was carried out against the gold standard, where a set of 938 manually reviewed cases where studied (178 HAIs/760 nonHAIs).

Globally, InNoCBR acting totally autonomous present a HAI sensitivity of 70.83% and a specificity of 97.76%, with a good positive predictive value of 77.24%. The kappa index for different type of infection is 0.67. Sensitivity varies depending on infection type, where bloodstream infection presents the best sensitivity (93.33%), whereas the respiratory is the infection type that could be improved the most (53.33%). As a semi-automatic system, taking InNoCBR's user final diagnosis, a high level of sensitivity (81.73%), specificity (99.47%) and, especially, PPV (94.33%) was obtained. This improvement of the semi-automatic evaluation with respect to the totally automatic comes mainly from correcting false negatives in respiratory infections. Moreover, this improvement in the overall accuracy is affordable, since InNoCBR users reported that confirming each new case with the aid of the diagnosis proposal, in combination with the user interface, which gives fast access to key patient information, is relatively fast.

Since 2013, the InNoCBR system has been deployed in more hospitals in Galicia (Spain), and now it is the standard system for HAI surveillance for the Galician public health system. The infection rates decreased from 5.53% (2014) to 4.06% (2018) in CHUO. Periodic reports generated with InNoCBR that are sent to the different medical services and nursing units in a monthly basis, along with comments and improvement proposals, led to this decrease in infection levels since 2013. Moreover, improvements in the infection prevention bundles have been included for several infection types, such as surgical site, bloodstream, among others. Finally, InNoCBR helped in suggesting changes in the antimicrobial therapy protocols for each type of infection, or even discourage an excessive antibiotic usage. Finally, the intelligent diagnostic module is being continuously auto-evaluated and reports the kappa index of a given period of time. For example, in the year 2018, the kappa index of concordance between the InNoCBR user and the InNoCBR proposals is 0.596, which is similar to that found during validation with 2013 data (kappa of 0.62, see Table 7).

Further directions of InNoCBR improvement could include (i) implementation of intelligent diagnosis for samples acquired in the pharmacy database, (ii) improvements of some filters in the acquisition module, and (iii) improvements of expert-provided, or automatically extracted classification rules.

## Figures and Tables

**Table 1:** 2 × 2 confusion matrix for a two-condition prediction system evaluation (TP = true positive, FP = false positive, FN = false negative, TN = true negative).

Prediction system	Gold standard	
	Positive	Negative
Positive	TP	FP
Negative	FN	TN

**Table 2:** *C* × *C* confusion matrix for a multiple-condition prediction system.

Prediction system	Gold standard							
	1	2	3	4	5	…	C	Total
1	X_11_	X_12_	X_13_	X_14_	X_15_	…	X_1C_	X_1_
2	X_21_	X_22_	X_23_	X_24_	X_25_	…	X_2C_	X_2_
3	X_31_	X_32_	X_33_	X_34_	X_35_	…	X_3C_	X_3_
4	X_41_	X_42_	X_43_	X_44_	X_45_	…	X_4C_	X_4_
5	X_51_	X_52_	X_53_	X_54_	X_55_	…	X_5C_	X_5_
…	…	…	…	…	…	…	…	…
C	X_C1_	X_C2_	X_C3_	X_C4_	X_C5_	…	X_CC_	X_C_
∑	X._1_	X_.2_	X_.3_	X_.4_	X_.5_	…	X._C_	n

**Table 3:** Gold standard descriptive analysis (U = Urinary infection, R = Respiratory infection, B = Bloodstream, S = Surgical site infection, C = Cutaneous infection, E = Enteric infection, O = Other type of infection, No/Ex=No infection or extrahospitalary infection).

Hospital unit	Type of infection								
	U	R	B	S	C	E	O	No/Ex	HAIs/∑
General surgery and Reanimation	34	11	8	33	—	2	2	285	90/375
Internal medicine	14	4	1	—	1	—	1	152	21/173
Nephrology	4	—	—	—	1	—	—	38	5/43
Traumatology	12	2	2	9	—	1	1	200	27/227
ICU	14	13	4	3	1	—	—	85	35/120
∑	78	30	15	45	3	3	4	760	178/938

**Table 4:** Global confusion matrix InNoCBR VS gold standard with different types of infection(U = Urinary infection, R = Respiratory infection, B = Bloodstream, S = Surgical site infection, C = Cutaneous infection, E = Enteric infection, O = Other type of infection, No/Ex = No infection or extrahospitalary infection). Neg^∗^ stands for any classification of InNoCBR different from a HAI: not acquired (indicated in parentheses), ignored, no infection or extrahospitalary infection.

InNoCBR	Infection type (gold standard)								
	U	R	B	S	C	E	O	No/Ex	∑
U	60	—	—	—	—	—	—	3	63
R	—	15	—	—	—	—	—	1	16
B	—	—	14	—	—	—	—	3	17
Q	—	—	—	40	—	—	2	7	49
C	—	—	—	—	1	—	—	3	4
E	—	—	—	—	—	1	—	1	2
O	—	—	—	—	—	—	—	—	0
Neg^∗^	18 (10)	14 (6)	1 (1)	5 (1)	2 (0)	2 (1)	2 (1)	743 (676)	787
∑	78	30	15	45	3	3	4	761	938

**Table 5:** Results obtained from the InNoCBR acquisition module when compared with the gold standard, for a 95% confidence interval.

Sensitivity	Specificity	PPV^∗^	NPV^∗^
88.76%	88.95%	46.26%	98.66%
(82.96–92.83)	(86.45–91.04)	(39.62–52.63)	(98.10–99.05)

^∗^Adjusted values for a prevalence of 9.68% (EPINE 2012).

**Table 6:** Acquired and not acquired HAIs by the InNoCBR acquisition module grouped by infection type (U = Urinary infection, R = Respiratory infection, B = Bloodstream, S = Surgical site infection, C = Cutaneous infection, E = Enteric infection, O = Other type of infection).

InNoCBR	Infection type (gold standard)							
	U	R	B	S	C	E	O	∑
Acquired	68	24	14	44	3	2	3	158
Not acquired	10	6	1	1	—	1	1	20
∑	78	30	15	45	3	3	4	178

**Table 7:** Results obtained from the InNoCBR intelligent diagnostic module when compared with the gold standard, for a 95% confidence interval.

Sensitivity^∗^ HAIs	Specificity^∗^ HAIs	PPV^∗^ HAIs	NPV^∗^ HAIs	kappa^∗^ index
81.06%	79.76%	77.24%	83.25%	0.62
(70.38–88.67)	(69.94–87.09)	(66.50–85.42)	(73.57–90.01)	(0.52–0.71)

^∗^Adjusted values for the following prevalences (EPINE 2012): urinary = 1.54%, respiratory = 1.97%, bloodstream = 1.38%, surgical = 2.61%, cutaneous = 0.31%, enteric = 0.15%, other = 1.72%.

**Table 8:** Global results obtained from InNoCBR when compared with the gold standard.

Sensitivity^∗^ HAIs	Specificity^∗^ HAIs	PPV^∗^ HAIs	NPV^∗^ HAIs	kappa^∗^ index
70.83%	97.76%	77.24%	76.00%	0.67
(60.21–79.6)	(96.46–98.61)	(66.50–85.42)	(72.96–78.80)	(0.60–0.74)

^*^Adjusted values for the following prevalences (EPINE 2012): urinary = 1.54%, respiratory = 1.97%, bloodstream = 1.38%, surgical = 2.61%, cutaneous = 0.31%, enteric = 0.15%, other = 1.72%.

**Table 9:** Confusion matrix, InNoCBR as a semi-automatic diagnostic system VS gold standard with different types of infection (U = Urinary infection, R = Respiratory infection, B = Bloodstream, S = Surgical site infection, C = Cutaneous infection, E = Enteric infection, O = Other type of infection, No/Ex = No infection or extrahospitalary infection).

User with InNoCBR	Infection type (gold standard)								
	U	R	B	S	C	E	O	No/Ex	∑
U	65	—	—	—	—	—	—	1	65
R	—	24	—	—	—	—	—	1	24
B	—	—	14	—	—	—	—	1	15
Q	—	—	—	43	—	—	—	—	43
C	—	—	—	—	3	—	—	—	3
E	—	—	—	—	—	2	—	—	2
O	—	—	—	—	—	—	2	1	3
Neg^∗^	13 (10)	6 (6)	1 (1)	2 (1)	0 (0)	1 (1)	2 (1)	756 (676)	781

∑	78	30	15	45	3	3	4	760	938

Neg^∗^: stands for any classification of InNoCBR different from a HAI: not acquired (indicated in parentheses), ignored, no infection or extrahospitalary infection.

**Table 10:** Global results obtained from InNoCBR as a semi-automatic diagnostic system VS gold standard.

Sensitivity^∗^ HAIs	Specificity^∗^ HAIs	PPV^∗^ HAIs	NPV^∗^ HAIs	kappa^∗^ index	kappa^∗^ index (only acquired)
81.73%	99.47%	94.33%	76.00%	0.87	0.91
(71.94–88.77)	(98.63–99.82)	(86.04–98.03)	(72.97–78.79)	(0.80–0.92)	(0.84–0.96)

^∗^Adjusted values for the following prevalences (EPINE 2012): urinary = 1.54%, respiratory = 1.97%, bloodstream = 1.38%, surgical = 2.61%, cutaneous = 0.31%, enteric = 0.15%, other = 1.72%.

**Table 11:** Different types of classification errors found in InNoCBR.

Process	Type of error	Count
Acquisition	False negative	20
	Not acquired	2
	Bad filtering of a pharmacy sample	13
	Bad filtering of a microbiology sample	5
Intelligent diagnostic	Pharmacy samples are not processed	15
	Different type of HAI	2
	False positive	17
	False negative	9

## Data Availability

The gold standard validation data used to support the findings of this study are restricted by the Galician (Spain) Research Ethics Committee in order to protect Patient Privacy.
